# Hepatic and Extrahepatic Insulin Clearance in Mice with Double Deletion of Glucagon-Like Peptide-1 and Glucose-Dependent Insulinotropic Polypeptide Receptors

**DOI:** 10.3390/biomedicines9080973

**Published:** 2021-08-06

**Authors:** Micaela Morettini, Agnese Piersanti, Laura Burattini, Giovanni Pacini, Christian Göbl, Bo Ahrén, Andrea Tura

**Affiliations:** 1 Department of Information Engineering, Università Politecnica delle Marche, 60131 Ancona, Italy; a.piersanti@pm.univpm.it (A.P.); l.burattini@univpm.it (L.B.); 2 Independent Researcher, 35142 Padova, Italy; giovannipacini49@gmail.com; 3 Department of Obstetrics and Gynecology, Medical University of Vienna, A-1090 Vienna, Austria; christian.goebl@meduniwien.ac.at; 4 Department of Clinical Sciences, Lund University, 221 84 Lund, Sweden; bo.ahren@med.lu.se; 5 Metabolic Unit, CNR Institute of Neuroscience, 35127 Padova, Italy; andrea.tura@cnr.it

**Keywords:** insulin clearance, incretin hormones, animal model, DIRKO, IVGTT, mathematical model

## Abstract

The aim of this study was to investigate whether incretins, at physiological levels, affect hepatic and/or extrahepatic insulin clearance. Hepatic and extrahepatic insulin clearance was studied in 31 double incretin receptor knockout (DIRKO) and 45 wild-type (WT) mice, which underwent an Intravenous Glucose Tolerance Test (IVGTT). A novel methodology based on mathematical modeling was designed to provide two sets of values (FE_L-P1_, CL_P-P1_; FE_L-P2_, CL_P-P2_) accounting for hepatic and extrahepatic clearance in the IVGTT first and second phases, respectively, plus the respective total clearances, CL_T-P1_ and CL_T-P2_. A statistically significant difference between DIRKO and WT was found in CL_T-P1_ (0.61 [0.48–0.82] vs. 0.51 [0.46–0.65] (median [interquartile range]); *p* = 0.02), which was reflected in the peripheral component, CL_P-P1_ (0.18 [0.13–0.27] vs. 0.15 [0.11–0.22]; *p* = 0.04), but not in the hepatic component, FE_L-P1_ (29.7 [26.7–34.9] vs. 28.9 [25.7–32.0]; *p* = 0.18). No difference was detected between DIRKO and WT in CL_T-P2_ (1.38 [1.13–1.75] vs. 1.69 [1.48–1.87]; *p* = 0.10), neither in CL_P-P2_ (0.72 [0.64–0.81] vs. 0.79 [0.69–0.87]; *p* = 0.27) nor in FE_L-P2_ (37.8 [35.1–43.1] vs. 39.8 [35.8–44.2]; *p* = 0.46). In conclusion, our findings suggest that the higher insulin clearance observed in DIRKO compared with WT during the IVGTT first phase may be due to its extrahepatic component.

## 1. Introduction

Glucagon-like peptide-1 (GLP-1) and glucose-dependent insulinotropic polypeptide (GIP) are incretin hormones responsible for the potentiation of insulin secretion following nutrient ingestion (i.e., the incretin effect) [1,2]. Such potentiation of insulin secretion is mainly accomplished by the expression of GLP-1 and GIP receptors on pancreatic β cells. In fact, due to its effect on insulin secretion, the incretin effect represents an important mechanism for the regulation of plasma insulin concentration. However, GLP-1 and GIP receptors are also expressed in other tissues, such as adipose, heart, kidney, bone, brain [3,4], and possibly liver [5] tissue, though the related functions remain mainly unknown [6].

It is well known that plasma insulin concentration is determined not only by the insulin secreted by the β cells but also by the insulin cleared from the plasma. Moreover, insulin clearance is a highly variable process, occurring mainly in the liver (i.e., hepatic insulin clearance) but also in other organs and tissues, mainly kidneys and skeletal muscles (i.e., extrahepatic insulin clearance). Some studies addressed the impact of the possible effect of incretins on total insulin clearance [7,8,9] or on its hepatic component [10,11], but in those studies, incretin hormones were administered at high pharmacological doses and showed controversial results. Only recently, the effect of physiological levels of incretins on insulin clearance was investigated in mice, showing that incretin hormones may reduce insulin clearance at non-stimulated levels [12]. However, whether this reduction involves hepatic and/or extrahepatic insulin clearance still remains unknown.

The aim of this study was therefore to investigate whether incretins at physiological, non-stimulated levels affect hepatic and/or extrahepatic insulin clearance. To this purpose, a mathematical modeling approach to segregate hepatic and extrahepatic insulin clearance was developed and applied to data from mice with the deletion of both the GLP-1 and GIP receptor genes (i.e., double incretin receptor knockout, DIRKO) [12] in comparison with their wild-type (WT) counterparts.

## 2. Materials and Methods

### 2.1. Animals

Hepatic and extrahepatic insulin clearance was studied in 31 double incretin receptor knockout (DIRKO) and 45 wild-type (WT) female mice and analyzed in a previous investigation [12]. The study was approved by the Lund/Malmö Animal Ethics Committee (approval No. M166-15, 23 October 2015). DIRKO (GLP-1R^−/−^:GIPR^−/−^) mice were generated from C57BL6J mice purchased from Taconic (Skensved, Denmark), according to a previously described procedure [13]. The GLP-1R^−/−^ mice were first backcrossed for five generations into the C57BL6J background. The GIPR^−/−^ mice were then crossed with the GLP-1R^−/−^ mice on the same C57BL6J background, after which subsequent heterozygotes were crossed to generate GLP-1R^−/−^:GIPR^−/−^ mice. Mice were maintained in a temperature-controlled room (22 °C) in a light–dark cycle of 12 h each (light on at 7:00 p.m.) and were fed with a standard pellet diet (Lactin, Stockholm, Sweden) and tap water ad libitum. During the experiment, food was removed from the cages at 7:30 a.m., and the actual experiments started at 12:30 a.m., i.e., during the dark cycle. After 5 h of fasting, the mice were anesthetized with an intraperitoneal injection of a fixed-dose combination of fentanyl (0.02 mg/mouse), fluanisone (0.5 mg/mouse; Vetpharma, Leeds, UK), and midazolam (0.125 mg/mouse; Roche, Basel, Switzerland). After 30 min, an Intravenous Glucose Tolerance Test (IVGTT) was performed. D-glucose (Sigma-Aldrich, Merck, Darmstadt, Germany) was injected in a tail vein at a dose of 0.35 g/kg. Blood samples (40 μL) were collected before and at 0, 1, 5, 10, 20, and 50 min after glucose administration. Plasma was immediately separated after collection and stored at –20 °C until analysis. Insulin concentration was determined by ELISA (Mercodia, Uppsala, Sweden). C-peptide concentration was determined by ELISA (Crystal Chem, Brook Drive, IL, USA). Glucose was measured using an Accu-Chek Aviva (Roche, Basel, Switzerland).

### 2.2. Assessment of Hepatic and Extrahepatic Insulin Clearance

**Figure 1:**
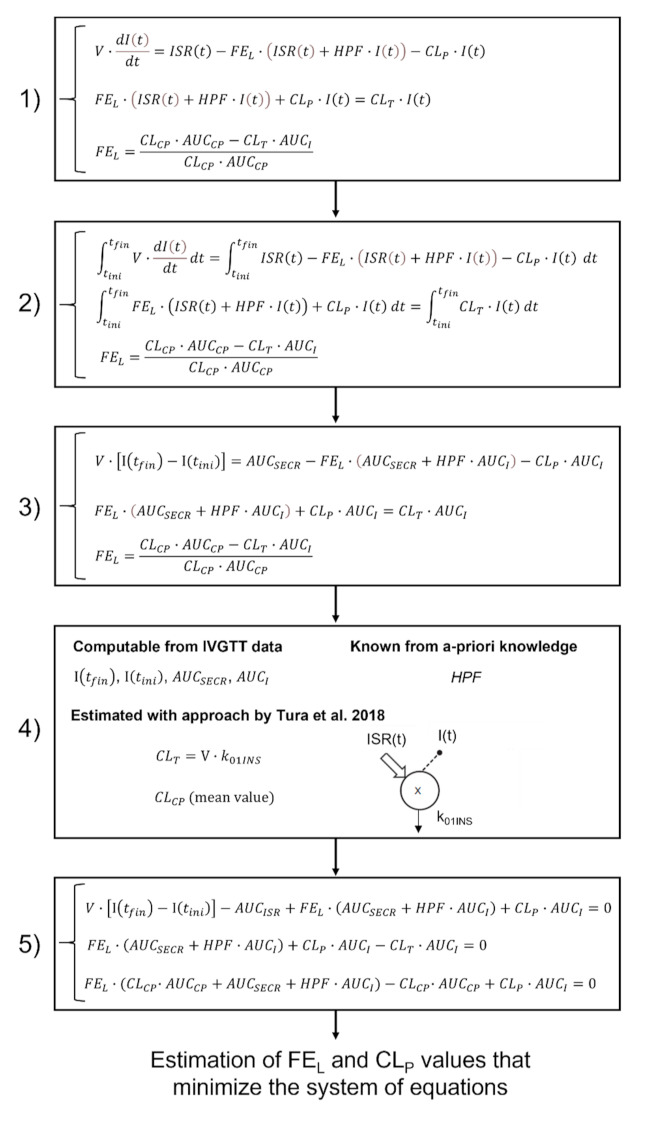
Flowchart of the methodology used to assess hepatic and extrahepatic insulin clearance. V: distribution volume; ISR(t): insulin secretion rate; FE_L_: hepatic insulin clearance; HPF(t): hepatic plasma flow rate; I(t): plasma insulin concentration; CL_P_: extrahepatic insulin clearance; CL_T_: total insulin clearance; CL_CP_: C-peptide clearance; AUC_CP_: area under the curve of C-peptide; AUC_I_: area under the curve of insulin; AUC_SECR_: area under the curve of ISR(t); t_ini_: initial time of IVGTT; t_fin_: final time of IVGTT.

A novel methodology inspired by previously proposed mathematical approaches was developed to assess hepatic and extrahepatic insulin clearance, as summarized in Figure 1.

In detail, according to the mathematical modeling approach proposed by Polidori et al. [14], the hepatic insulin clearance rate, HICR(t) (pmol/min), was assumed linear with respect to insulin delivery to the liver:

(1) $$HICR\left(t\right)=FE_{L}\cdot \left(ISR\left(t\right)+HPF\cdot I\left(t\right)\right)$$

where FE_L_ is the hepatic fractional extraction (dimensionless), ISR(t) is the insulin secretion rate (pmol/min), HPF is the hepatic plasma flow (l/min), and I(t) is the plasma insulin concentration (pmol/l); extrahepatic insulin clearance rate, EICR(t) (pmol/min), is assumed proportional to the plasma insulin concentration:

(2) $$EICR\left(t\right)=CL_{P}\cdot I\left(t\right)$$

where CL_P_ is the extrahepatic insulin clearance (l/min). Thus, changes in the plasma insulin compartment (characterized by the distribution volume V) can be described as follows:

(3) $$V\cdot \frac{dI\left(t\right)}{dt}=ISR\left(t\right)-FE_{L}\cdot \left(ISR\left(t\right)+HPF\cdot I\left(t\right)\right)-CL_{P}\cdot I\left(t\right)$$

Moreover, total insulin clearance rate can be described as the sum of the hepatic and extrahepatic insulin clearance rates:

(4) $$FE_{L}\cdot \left(ISR\left(t\right)+HPF\cdot I\left(t\right)\right)+CL_{P}\cdot I\left(t\right)=CL_{T}\cdot I\left(t\right)$$

where CL_T_ is the total insulin clearance (l/min) and I(t) is the plasma insulin concentration measured during the IVGTT. ISR(t) was computed according to Van Cauter et al. [15] by deconvolution from the plasma C-peptide concentration using individualized C-peptide kinetic parameters.

Equations (3) and (4) were considered simultaneously (step 1, Figure 1), and their temporal integral was calculated between the initial and final time points of the IVGTT, indicated as t_ini_ and t_fin_, respectively (step 2, Figure 1). Integration provides two algebraic equations (step 3, Figure 1) in which I(t_ini_), I(t_fin_), AUC_SECR_, and AUC_I_ are quantities computable from the IVGTT data (step 4, Figure 1), representing plasma insulin concentration at the IVGTT initial and final time points, and the area under the curve of ISR(t) and I(t), respectively. CL_T_ and V were estimated using the mathematical modeling approach proposed by Tura et al. [12] (step 4, Figure 1); this approach assumes a mono-compartmental description for insulin kinetics, where ISR(t) and I(t) represents the input and the output of the model, respectively. HPF has been assumed equal to 2 l/h/kg of body weight [16]. A third equation taken from [17] and describing hepatic fractional extraction, FE_L_, was considered in addition to the previous two:

(5) $$FE_{L}=\left(AUC_{CP}\cdot CL_{CP}-AUC_{I}\cdot CL_{T}\;\right)/\;\left(AUC_{CP}\cdot CL_{CP}\right)$$

where AUC_CP_ is the area under the curve of C-peptide and CL_CP_ (l/min) is the C-peptide clearance. The three algebraic equations, written as a homogeneous system (step 5, Figure 1), were used as elements of a cost function, which was minimized to obtain estimates of FE_L_ and CL_P_. The procedure was implemented in MATLAB^®^ R2017b (MathWorks, Natick, MA, USA), and the minimum cost function was obtained using the *lsqnonlin* function.

The methodology summarized in Figure 1 was used to estimate the two pairs of values (FE_L-P1_, CL_P-P1_, and FE_L-P2_, CL_P-P2_) accounting for hepatic and extrahepatic clearance in the IVGTT first and second phases, respectively. The first pair (FE_L-P1_, CL_P-P1_) was estimated assuming t_ini_ = 0 min and t_fin_ = 5 min; the second pair (FE_L-P2_; CL_P-P2_) was estimated assuming t_ini_ = 5 min and t_fin_ = 50 min. The corresponding values of insulin and C-peptide plasma concentrations were used for the estimations. Consistently, the total clearance was estimated for the first and second phases and denoted as CL_T-P1_ and CL_T-P2_, respectively.

### 2.3. Assessment of Other Parameters of Glucose Metabolism

A detailed metabolic assessment was obtained by assessing: (i) the glucose tolerance, as the K_G_ index [18]; (ii) the acute insulin response to glucose (AIR_G_), as the average suprabasal insulin between 1 and 5 min after glucose administration; (iii) the insulin sensitivity during the IVGTT (S_I_), as an empirical parameter predicting insulin sensitivity from the minimal model approach [18,19]); (iv) the insulin sensitivity at fasting, as QUICKI [20]; (v) the glucose effectiveness (S_G_) [18,19]; (vi) the disposition index, as a product between S_I_ and AIR_G_, similar to that in human subjects [21]; (vii) the β-cell function, as a ratio of the area under the curve of C-peptide to that of glucose, extending the approach used in human subjects [22]; and (viii) the area under the curve of the insulin secretion rate (AUC_SECR_) calculated as previously indicated according to Van Cauter et al. [15].

### 2.4. Statistical Analysis

The Shapiro–Wilk test showed a skewed (i.e., not normal) distribution of variables. Thus, the variables were presented as median [interquartile range (IQR)]. Differences in variable mean values between the two groups were tested by an unpaired t-test. A univariable linear regression analysis was used to assess the associations between insulin clearance and the other metabolic parameters. Tests were applied to the log-transformed values. The two-sided significance level was set at 5% (*p* < 0.05).

## 3. Results

The values for hepatic, extrahepatic, and total insulin clearance in the first and second phases of the IVGTT are reported in Table 1 for both DIRKO and WT mice.

A significant difference between DIRKO and WT was found in CL_T-P1_, which was reflected in the peripheral (i.e., extrahepatic) component, CL_P-P1_, but not in the hepatic component, FE_L-P1_. No difference was detected in CL_T-P2_ in any of its components, that is, neither in CL_P-P2_ nor in FE_L-P2_. In addition, the ratio between the two insulin clearance components was statistically different between the two groups for the first phase (*p* = 0.03) but not for the second phase (*p* = 0.30).

A detailed IVGTT-based metabolic assessment for DIRKO and WT mice is reported in Table 2. Statistically significant differences were detected in the IVGTT-based glucose tolerance index, K_G_, in insulin sensitivity during the IVGTT, S_I_; in glucose effectiveness, S_G_; in the disposition index, DI; and in the area under the curve of the insulin secretion rate calculated from C-peptide, AUC_SECR_. β-cell sensitivity (ratio of the area under the curve of C-peptide to that of glucose) showed borderline *p*-values. Insulin sensitivity at fasting, QUICKI, was not different. 

**Figure 2:**
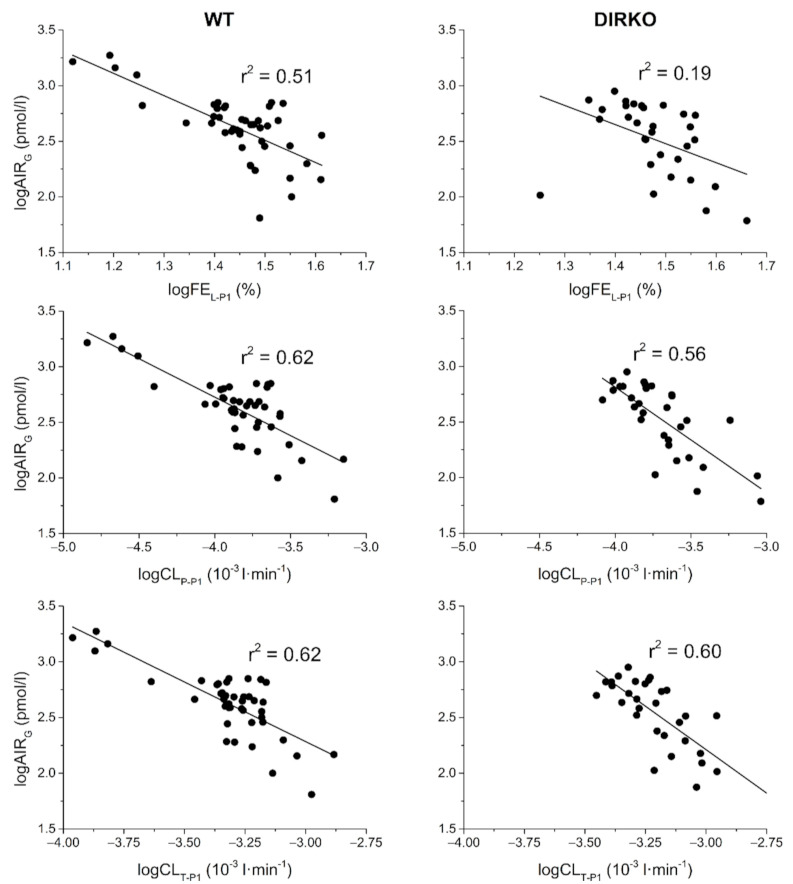
Most significant associations (r^2^) of hepatic, extrahepatic, and total insulin clearance with metabolic parameters in DIRKO and WT mice. FE_L-P1_, CL_P-P1_, and CL_T-P1_: hepatic, extrahepatic, and total clearance during the first phase of the test, respectively; AIR_G_: acute insulin response to glucose.

The results of the linear regression analysis to evaluate associations between insulin clearance and the other metabolic parameters are reported in Table 3. The most significant associations are reported in Figure 2.

## 4. Discussion

This study investigated whether insulin clearance reduction, previously observed in DIRKO compared with WT mice under non-stimulated levels of incretin hormones [12], is explained by a reduction in its hepatic or extrahepatic component, or both. DIRKO is a mouse model already exploited in previous studies and characterized by the knockout of both the GLP-1 and GIP receptors. In contrast with single incretin receptor knockout mice, knockout of both incretin receptors prevents the possibility that one hormone compensates for the lack of biological action of the other. The phenotypic traits of DIRKO with respect to WT include lower number of pancreatic islets (though not pancreatic mass) [23], impaired insulin secretion [13], reduced circulating GIP (but not GLP-1) and catecholamines levels after oral glucose [12], lack of the incretin effect [24], reduced glucose tolerance, [25] and reduced insulin sensitivity [26]. As shown in the results of this study, DIRKO mice are also characterized by reduced glucose effectiveness and the ability of β-cells to compensate for insulin resistance (i.e., reduced disposition index despite higher secretion during the IVGTT).

In our study, the hepatic component of insulin clearance, as quantified by the FE_L_ parameter, in both the first and second phases of the IVGTT, did not show differences in DIRKO compared with WT mice. This observation can be analyzed in the light of the mechanisms and physiology of hepatic insulin clearance, in particular with reference to the role of carcinoembryonic antigen-related cell adhesion molecule 1 (CEACAM1). As recently reviewed in [27], evidence showed that CEACAM1 participates in the insulin–insulin receptor complex to increase the rate of its endocytosis and targeting to the degradation pathways by coordinating its effect with the insulin-degrading enzyme (IDE), the most-abundant protease that degrades insulin in the cytosol. On the other hand, it has been shown that acute insulin pulses during the first few hours of refeeding (following an overnight fast) induce the transcriptional activity of the CEACAM1 promoter to stimulate CEACAM1 expression [27,28], and through this, insulin stimulates its own clearance in hepatocytes. In our study, FE_L_ during the first phase is significantly correlated with first-phase insulin secretion (i.e., AIR_G_), but no difference has been detected between DIRKO and WT mice in AIR_G_, thus suggesting unaltered CEACAM1 expression in the liver. This may explain the lack of difference between the two groups in the hepatic insulin clearance.

It should be noted that previous studies showed that exenatide, a glucagon-like peptide-1 receptor agonist, promotes insulin clearance by inducing the expression of CEACAM1 in hepatocytes [29]. The absence of differences that we observed between DIRKO and WT mice in the hepatic insulin clearance could be ascribed to the fact that our study was performed under non-stimulated levels of incretin hormones, and hence, the level of GLP-1 in WT may be insufficient to increase the CEACAM1 expression compared with DIRKO.

The most interesting finding of this study is that, during the first phase of the IVGTT, total insulin clearance is higher in DIRKO than in WT, and this may be due to the higher extrahepatic component. Our study did not allow for the elucidation of the factors involved in extrahepatic insulin clearance regulation. What is known is that kidneys are the primary site of extrahepatic insulin clearance [30] and that different pathways can be involved in its regulation, as recently demonstrated [31]. Insulin is cleared in the proximal tubule cells of the kidneys by two major routes, either by absorption of filtered insulin or by post-glomerular capillary secretion. Only a minor portion appears to be excreted in urine. IDE is the major enzyme responsible for degrading insulin in the kidneys, and novel findings have shown that its expression in human and rodent kidneys is regulated by sorting nexin 5 (SNX5), a cytoplasmic and a membrane-associated protein belonging to the SNX family that regulates intracellular trafficking in the brush-border membrane of proximal tubules [32]. Of note, SNXs can play an important role in the regulation of insulin metabolism. Indeed, SNXs have been shown to control β-cell incretin responses by regulating the balance between GLP-1 receptor plasma membrane recycling and lysosomal degradation [33]. Since WT mice express GLP-1 receptors, we can hypothesize that the difference observed in the extrahepatic insulin clearance can be linked to the indicated mechanism involving SNXs. This, however, needs to be elucidated in future studies.

With regard to GIP, little is known about its possible role in insulin clearance regulation, but the few published studies support the idea that GIP does not affect insulin clearance [7]. However, due to their importance in determining insulin and glucose metabolism, molecular mechanisms involving GIP deserve further investigation.

It should be acknowledged that the DIRKO mouse model has a limitation, since it does not allow for the dissection of possible separate effects of GLP-1 and GIP. However, the main findings in one of our previous studies on single incretin hormone knockout suggested a lack of effect for both GLP-1 and GIP when acting in isolation [34]. Thus, in the present analysis, which is aimed at determining possible different aspects of hepatic and extrahepatic insulin clearance, we considered the study of the DIRKO mouse model as more appropriate for our purposes. In addition, as briefly mentioned before, the incretin single knockout models do not show ideal behavior, since compensatory phenomena may arise (e.g., It appears that deletion of one incretin hormone receptor leads to increased expression of the other receptor [12]).

To segregate the hepatic and extrahepatic components of insulin clearance, a mathematical modeling approach has been developed. The proposed mathematical modeling approach exploited those previously proposed by Polidori et al. [14], adding to the model equations other independent equations that linked hepatic and extrahepatic components to the total insulin clearance. Of note, Polidori’s approach, which is based on a single differential equation, was conceived to work with insulin-modified IVGTT, as this kind of test allows for better separation of the two insulin clearance components. Thus, data derived by tests with slower dynamics, such as oral glucose tolerance tests, may not be adequate to be analyzed with the modeling approach proposed in this study for separate assessment of the hepatic and extrahepatic insulin clearance components, as the model parameters may be estimated with insufficient accuracy. In addition, it should be observed that, in humans (women with a history of gestational diabetes), insulin clearance derived by an insulin-modified intravenous glucose tolerance test was found to be more relevant than that derived by an oral glucose tolerance test for the assessment of type 2 diabetes risk [35]. On the other hand, it has to be noted that, in [35], only total clearance was analyzed, without dissection into its hepatic and extrahepatic components.

In a previous study, it was shown that glucagon may be inversely related to insulin clearance [36]. It is also worth noting that, in our previous study on incretin hormone single knockouts, we found different glucagon secretion levels depending on the deleted incretin hormone receptor [34]. Unfortunately, in our DIRKO mice, glucagon data are not available, and thus, this has to be acknowledged as a limitation of the present analysis and as a factor to possibly be addressed in future studies.

## 5. Conclusions

A mathematical approach to differentiate hepatic and extrahepatic insulin clearance during an IVGTT was developed. We found that, during the first phase of the test, the higher total insulin clearance observed in DIRKO compared with WT mice may be due to the higher extrahepatic component. These findings may help to shed light on understanding the mechanisms of insulin clearance and their possible role in the pathogenesis of type 2 diabetes. In fact, knowledge of the relative importance of hepatic versus extrahepatic components of insulin clearance and their relationship with the incretin system may be of help in the development of therapeutic agents for type 2 diabetes, especially in addressing possible defects in the balance between these mechanisms.

## Figures and Tables

**Table 1:** Values for hepatic, extrahepatic, and total insulin clearance in the IVGTT first and second phases for DIRKO and WT mice.

		WT	DIRKO	*p*-Value
First phase	FE_L-P1_ (%)	28.9 [25.7–32.0]	29.7 [26.7–34.9]	0.18
	CL_P-P1_ (10^−3^ l∙min^−1^)	0.15 [0.11–0.22]	0.18 [0.13–0.27] *	0.04
	CL_T-P1_ (10^−3^ l∙min^−1^)	0.51 [0.46–0.65]	0.61 [0.48–0.82] *	0.02
Second phase	FE_L-P2_ (%)	39.8 [35.8–44.2]	37.8 [35.1–43.1]	0.46
	CL_P-P2_ (10^−3^ l∙min^−1^)	0.79 [0.69–0.87]	0.72 [0.64–0.81]	0.27
	CL_T-P2_ (10^−3^ l∙min^−1^)	1.69 [1.48–1.87]	1.38 [1.13–1.75]	0.10

The data are reported as median [interquartile range]; FE_L-P1_, CL_P-P1_, and CL_T-P1_: hepatic, extrahepatic, and total insulin clearance in the IVGTT first phase, respectively; FE_L-P2_, CL_P-P2_, and CL_T-P2_: hepatic, extrahepatic, and total insulin clearance in the IVGTT second phase, respectively; * *p* < 0.05 DIRKO vs. WT.

**Table 2:** Metabolic assessment for DIRKO and WT mice.

	WT	DIRKO	*p*-Value
K_G_ (%/min)	2.49 [1.85–3.14]	1.98 [0.96–2.41] *	<0.01
S_I_ (10^−4^ min^−1^/pmol/l)	1.37 [0.97–1.78]	0.85 [0.77–1.39] *	<0.01
QUICKI (dimensionless)	0.123 [0.119–0.126]	0.120 [0.116–0.123]	0.07
S_G_ (min^−1^)	0.052 [0.037–0.063]	0.037 [0.028–0.055] *	0.02
AIR_G_ (pmol/l)	445 [288–635]	426 [195–634]	0.16
DI (10^−3^ min^−1^)	1.17 [0.70–1.64]	0.72 [0.34–1.03] *	0.02
AUC_SECR_ (pmol)	13.0 [12.0–16.8]	17.0 [14.3–21.0] *	<0.01
β-cell sensitivity (mmol C-peptide/mmol Glucose)	0.022 [0.018–0.027]	0.025 [0.021–0.030]	0.05

The data are reported as median [interquartile range]; K_G_: intravenous glucose tolerance index; S_I_: insulin sensitivity during the IVGTT; QUICKI: insulin sensitivity at fasting; S_G_: glucose effectiveness; AIR_G_: acute insulin response to glucose; DI: disposition index; AUC_SECR_: area under the curve of insulin secretion rate; β-cell sensitivity: ratio of the area under the curve of C-peptide to the area under the curve of glucose; * *p* < 0.05 DIRKO vs. WT.

**Table 3:** Association between insulin clearance and the other metabolic parameters.

		First Phase			Second Phase		
		FE_L-P1_	CL_P-P1_	CL_T-P1_	FE_L-P2_	CL_P-P2_	CL_T-P2_
WT + DIRKO	K_G_	n.s.	0.08 (0.01)	0.08 (0.01)	0.08 (0.01)	n.s.	0.07 (0.03)
	S_I_	n.s.	n.s.	n.s.	n.s.	n.s.	n.s.
	QUICKI	0.06 (0.02)	0.10 (<0.01)	0.12 (<0.01)	n.s.	n.s.	n.s.
	S_G_	n.s.	n.s.	n.s.	0.06 (0.03)	n.s.	n.s.
	AIR_G_	0.37 (<0.001)	0.59 (<0.001)	0.60 (<0.001)	n.s.	n.s.	n.s.
	DI	0.23 (<0.001)	0.38 (<0.001)	0.39 (<0.01)	0.07 (0.02)	n.s.	n.s.
	AUC_SECR_	0.07 (0.02)	0.13 (<0.01)	0.12 (<0.01)	0.17 (<0.001)	n.s.	n.s.
	β-cell sensitivity	0.06 (0.03)	0.11 (<0.01)	0.11 (<0.01)	n.s.	n.s.	0.11 (<0.01)
WT	K_G_	n.s.	n.s.	n.s.	n.s.	n.s.	n.s.
	S_I_	n.s.	n.s.	n.s.	n.s.	n.s.	n.s.
	QUICKI	n.s.	n.s.	n.s.	n.s.	n.s.	n.s.
	S_G_	n.s.	n.s.	n.s.	n.s.	n.s.	n.s.
	AIR_G_	0.51 (<0.001)	0.62 (<0.001)	0.62 (<0.001)	n.s.	n.s.	n.s.
	DI	0.33 (<0.001)	0.40 (<0.001)	0.40 (<0.001)	n.s.	n.s.	n.s.
	AUC_SECR_	0.15 (<0.01)	0.14 (0.01)	0.15 (<0.01)	0.15 (<0.01)	n.s.	n.s.
	β-cell sensitivity	0.16 (<0.01)	0.22 (<0.01)	0.23 (<0.001)	n.s.	n.s.	0.11 (0.03)
DIRKO	K_G_	n.s.	n.s.	n.s.	n.s.	n.s.	0.14 (0.04)
	S_I_	n.s.	n.s.	n.s.	n.s.	n.s.	n.s.
	QUICKI	n.s.	n.s.	n.s.	n.s.	n.s.	n.s.
	S_G_	n.s.	n.s.	n.s.	n.s.	n.s.	n.s.
	AIR_G_	0.19 (0.01)	0.56 (<0.001)	0.60 (<0.001)	n.s.	n.s.	0.19 (0.01)
	DI	n.s.	0.29 (<0.01)	0.31 (<0.01)	n.s.	n.s.	0.26 (<0.01)
	AUC_SECR_	n.s.	n.s.	n.s.	0.37 (<0.001)	n.s.	0.15 (0.03)
	β-cell sensitivity	n.s.	n.s.	n.s.	n.s.	n.s.	n.s.

For each statistically significant association, the r^2^ (*p*-value) is reported; n.s.: not significant association. FE_L-P1_, CL_P-P1_, CL_T-P1_, FE_L-P2_, CL_P-P2_, and CL_T-P2_: hepatic, extrahepatic, and total clearance during the first and second phases of the test, respectively; K_G_: intravenous glucose tolerance index; S_I_: insulin sensitivity during the IVGTT; QUICKI: insulin sensitivity at fasting; S_G_: glucose effectiveness; AIR_G_: acute insulin response to glucose; DI: disposition index; AUC_SECR_: area under the curve of the insulin secretion rate; β-cell sensitivity: ratio of the area under to curve of C-peptide to the area under the curve of glucose.

## Data Availability

The data presented in this study are available from Prof. Bo Ahrén upon reasonable request.

## Abbreviations

AIR_G_	Acute Insulin Response to glucose
AUC_CP_	Area Under the Curve of C-peptide
AUC_I_	Area Under the Curve of insulin
AUC_SECR_	Area Under the Curve of insulin secretion rate
CEACAM1	Carcinoembryonic Antigen-related Cell Adhesion Molecule 1
CL_CP_	C-peptide clearance during IVGTT
CL_P-P1_	Extrahepatic insulin clearance in the first phase of IVGTT
CL_P-P2_	Extrahepatic insulin clearance in the second phase of IVGTT
CL_T-P1_	Total insulin clearance in the first phase of IVGTT
CL_T-P2_	Total insulin clearance in the second phase of IVGTT
DI	Disposition Index
DIRKO	Double Incretin Receptor KnockOut
EICR(t)	Extrahepatic Insulin Clearance Rate
FE_L-P1_	Hepatic insulin clearance in the first phase of IVGTT
FE_L-P2_	Hepatic insulin clearance in the second phase of IVGTT
GIP	Glucose-dependent insulinotropic polypeptide
GLP-1	Glucagon-like Peptide 1
HICR(t)	Hepatic Insulin Clearance Rate
HPF	Hepatic Plasma Flow
I(t)	Plasma insulin concentration
IDE	Insulin-Degrading Enzyme
ISR(t)	Insulin Secretion Rate
IVGTT	Intravenous Glucose Tolerance Test
K_G_	Glucose tolerance
QUICKI	Quantitative Insulin Sensitivity Check Index
S_I_	Insulin sensitivity during the IVGTT
S_G_	Glucose effectiveness
SNX	Sorting Nexin
t_fin_	Final time of IVGTT
t_ini_	Initial time of IVGTT
V	Distribution volume
WT	Wild-Type
